# Physico-Chemical Properties and Texturization of Pea, Wheat and Soy Proteins Using Extrusion and Their Application in Plant-Based Meat

**DOI:** 10.3390/foods12081586

**Published:** 2023-04-08

**Authors:** Delaney Webb, Hulya Dogan, Yonghui Li, Sajid Alavi

**Affiliations:** Department of Grain Science and Industry, Kansas State University, Manhattan, KS 66506, USA

**Keywords:** plant protein, functional properties, texturization, phase transition, meat analogues

## Abstract

Four commercial pea protein isolates were analyzed for their physico-chemical properties including water absorption capacity (WAC), least gelation concentration (LGC), rapid visco analyzer (RVA) pasting, differential scanning calorimetry (DSC)-based heat-induced denaturation and phase transition (PTA) flow temperature. The proteins were also extruded using pilot-scale twin-screw extrusion with relatively low process moisture to create texturized plant-based meat analog products. Wheat-gluten- and soy-protein-based formulations were similarly analyzed, with the intent to study difference between protein types (pea, wheat and soy). Proteins with a high WAC also had cold-swelling properties, high LGC, low PTA flow temperature and were most soluble in non-reducing SDS-PAGE. These proteins had the highest cross-linking potential, required the least specific mechanical energy during extrusion and led to a porous and less layered texturized internal structure. The formulation containing soy protein isolate and most pea proteins were in this category, although there were notable differences within the latter depending on the commercial source. On the other hand, soy-protein-concentrate- and wheat-gluten-based formulations had almost contrary functional properties and extrusion characteristics, with a dense, layered extrudate structure due to their heat-swelling and/or low cold-swelling characteristics. The textural properties (hardness, chewiness and springiness) of the hydrated ground product and patties also varied depending on protein functionality. With a plethora of plant protein options for texturization, understanding and relating the differences in raw material properties to the corresponding extruded product quality can help tailor formulations and accelerate the development and design of plant-based meat with the desired textural qualities.

## 1. Introduction

The popularity of plant-based meat is soaring. A global interest in consuming protein sources which are perceived as ethical has been fostered in recent years, causing the rising interest in plant-based meat alternatives [1]. In the US, the increase in sales proves this; the plant-based meat market grew to USD 4.2 billion in 2020, a 24% increase from 2019 [2].

There are several types of plant-based meat including the traditional, gluten-based product seitan and newer extruded forms [3]. Among the latter, products that are extruded at a relatively low moisture level (30–40% wet basis) in order to generate the requisite mechanical energy in the process for protein cooking and cross-linking can be grouped together broadly as texturized vegetable protein. Although having fibrous layers like meat, these products are porous and are often further processed via milling, drying and/or rehydration before use [4,5]. Another category of extruded products, called high moisture meat analogs or HMMA, are processed at a much higher moisture level (example, 60–65% wet basis) in order to have a meat-like texture without the need for much further processing, and rely on a long slit die for cooling, layering and densification at the discharge end of the extruder [3,5]. While both single- and twin-screw technologies can be used to make extruded plant-based meat, the HMMA products rely on the latter for the pumping capacity to move the product through the cooling die.

The plant-based market in 2019 relied heavily on soy, with soy-based products making up 48% of the plant-based meat market [6]. However, there is growing commercial investment in pea protein and large entities, including McDonald’s and Beyond Meat besides many other companies, are entering this unique space. Thus, this alternative protein source is fast growing its market share [7]. Greenhouse gas emissions and land usage for production of pea protein are much lower (0.4 kg CO_2_eq and 3.4 m^2^, respectively, per 100 g) than what is required for beef (50 CO_2_eq and 164 m^2^ per 100 g), contributing to environmental ethics considerations and the rise of plant-based meats with pea ingredients [8,9]. Additionally, pea protein is attractive for companies to fulfill consumer desires for cleaner labels, as pea protein has low allergenicity and is non-GMO [9].

Although many companies use pea protein, different ingredient sources are associated with vastly different agronomic, isolation and commercial production conditions, leading to different raw material functionality [10]. Pea protein can be extracted in a wet environment or through dry fractionation and air classification. The more commonly used wet extraction process involves soaking yellow peas in water, crushing them, and separating the fiber and starch. The remaining protein is then placed in an alkaline solution for neutralization and extraction through the isoelectric point and then steam sterilized before being spray dried [11,12]. Isolating at a pH of 9 increases the aggregation of protein, decreases protein solubility and also the beaniness of the isolate compared to isolating at a pH of 8.5 [13]. A small adjustment in processing clearly leads to different protein functions.

For functional purposes, pea protein isolates may also be hydrolyzed with papain or bromelain enzymes or cross-linked with transglutaminase [11]. Some initial steps with the fermentation of pea protein for functional purposes have also been taken, though they have not been commercialized yet [14]. Prolonged heat treatment or exposure to high temperatures denatures the protein [15]. With varying heat and pH treatments, milling parameters, and hydrolysis, it is obvious that the function of pea proteins would vary greatly among suppliers. All of these differences are specific to the isolation process and are in addition to the differences that may exist prior to the isolation due to cultivar and environmental variances [16,17].

The goal of this study was to determine the raw material physico-chemical characteristics of multiple commercial pea protein isolates (water absorption, heat gelation, denaturation qualities, viscosity) and determine how those qualities may create unique opportunities for the extrusion-based textured product traits (water holding capacity, bulk density, layering, hardness, etc.) as well as to understand the relationships between protein properties and the internal structure of final product. The primary hypothesis was that protein physico-chemical properties, especially relating to hydration characteristics, can be an important determinant of end-product quality including cross-linking and layering. Soy and wheat gluten proteins, the conventional raw materials in plant-based meat, were also studied for comparison with pea proteins. The chemistry and functionality of these proteins have been reviewed previously [10]. Legumes such as soy and pea have a high concentration of globulin proteins, although the ratio of their legumin and vicilin fractions vary and that can dictate their functionality significantly. On the other hand, wheat gluten is mostly comprised of gliadin and glutenin proteins, leading to its unique properties. A relatively low moisture (30–40% wet basis) extrusion process, as described above, was used to texturize these proteins, with the products intended for longer storage before hydration for plant-based meat applications.

## 2. Materials and Methods

### 2.1. Materials

The purpose was to compare different commercial pea proteins, as well as compare different types of proteins. The plant proteins were selected based on market presence. Pea protein isolate was sourced from four separate companies (PP1–PP4). Soy protein isolate and soy protein concentrate (SPC) were also obtained from a commercial source. Vital wheat gluten (VWG) was obtained from MGP Ingredients (Atchison, KS, USA) and hard red winter wheat flour was obtained from Hal Ross Flour Mill (Kansas State University, Manhattan, KS, USA).

A total of 8 treatments were tested in this study: 4 pea treatments, 2 wheat treatments, and 2 soy treatments. Treatment formulations are described in Table 1 and were created on the basis of protein content and prior knowledge of each protein to create a viable product. The composition of the main treatments of interest is shown in Table 2. Pea protein isolates had a protein content ranging from 80–83% db. VWG had a higher level of protein (86.7%), while the protein content of SPC was lower (70%). Wheat Mix and Soy Mix treatments were included to match the level of protein in the pea protein isolates to facilitate better comparison. For the Wheat Mix treatment, vital wheat gluten was diluted to roughly 80% protein with wheat flour, which had 12.5% protein, while in the Soy Mix treatment, soy protein isolate with 90% protein was added to SPC to increase the protein content to roughly 78%. The range of protein content of the final formulations (77.8–86.7%) was designed to be similar to that of animal meats such as chicken, fish and beef on a dry basis (77.7–86.7%) [18].

### 2.2. Extrusion Parameters and Calculations

A ribbon blender (Wenger Manufacturing, Sabetha, KS, USA) was used to mix the soy and wheat treatments for 5 min. A pilot-scale (52 mm diameter, L/D ratio of 19.5), co-rotating twin-screw extruder (Model TX-52, Wenger Manufacturing, Sabetha, KS, USA) was used for texturization. Operating parameters for each treatment can be found in Table 3. The dry material feed rate was constant for all pea and wheat protein treatments at 50 kg/h. The dry feed rate was decreased to 40–45 kg/h for the soy treatments. The extruder screw speed was 450 rpm for pea and wheat treatments and 200–320 rpm for soy treatments. Water was added at a rate of 8 kg/h in the preconditioner for all treatments. Pea protein treatments received water in the extruder barrel at 8 kg/h, but wheat and soy treatments required 12–14 kg/h. A lower feed rate and/or screw speed, and also higher water input was required for wheat and soy protein treatments as described above because they tend to need less energy for texturization. High extrusion mechanical energy often leads to a less than optimal product for these two protein types, and in the case of soy, burning. Steam injection was not used in any of the treatments. Four temperature zones were used at 40, 70, 90 and 110 °C from the inlet of the extruder barrel to the outlet.

The screw profile was composed of double flighted elements of decreasing pitch, with two forward kneading blocks and four reverse kneading blocks dispersed throughout the profile, and a conical cut element at the end (Table 4). A 1/8” (3.172 mm) venturi die (or back die) was used for all treatments, except soy treatments that used a ¼” (6.35 mm) venturi or back die to prevent burning. After the venturi die, a 11” (27.94 cm) long Teflon spacer was placed, and then the final die plate which had two ¼” (6.35 mm) final circular die openings. Three hard knives were used with a knife speed of 250 rpm to cut the product. A sample was taken from the extruder, immediately milled to 0.18” (4.6 mm) pieces (Comitrol, Urschel Laboratories Incorporated, Valparaiso, IN, USA) and frozen. Whole extrudate samples were dried at 200 °C for 12 min and cooled for 8 min in a dual-pass drier (Series 4800, Wenger Manufacturing, Sabetha, KS, USA). Dried extrudate samples were stored at room temperature.

Specific mechanical energy (SME) was calculated using the following formula:

(1) $$SME\left(\frac{kJ}{kg}\right)=\frac{\left(\frac{\tau -\tau_{0}}{100}\right)\times \frac{N}{N_{r}}\times P_{r}}{m_{f}}$$

where τ is the % torque, τ_0_ is the no-load torque %, N is the measured screw speed in rpm, N_r_ is the rated screw speed (336 rpm), P_r_ is the rated motor power (22.4 kW) and m_f_ is mass flow rate in kg/s.

In-barrel moisture (IBM) content was calculated using the following equation:

(2) $$IBM\left(\%wb\right)=\frac{\left(m_{f}\times X_{wf}\right)+m_{wp}+m_{we}}{m_{f}+m_{wp}+m_{we}}$$

where m_f_ is the dry feed rate, X_wf_ is the moisture content of the dry feed material (expressed as wet basis fraction), m_wp_ is the water injection rate into the pre-conditioner in kg/h and m_we_ is the water injection rate into the extruder in kg/h. An IBM of roughly 29% was used for pea protein treatments, while 35–38% IBM was used for wheat and soy treatments.

### 2.3. Moisture Content

Moisture content was measured for raw ingredients, preconditioned treatments and extrudates (before drying), using the AACC 44–19.01 method. Triplicate samples of approximately 2 g were dried at 135 °C for 2 h for this procedure.

### 2.4. Raw Material Analysis

#### 2.4.1. Particle Size Distribution

The particle size distribution of each treatment was determined in duplicate using an Air Jet Sieve e200LS (Hosokawa Alpine Group, Augsburg, Germany). A 100 g sample was placed on the smallest sieve with a negative pressure of 3400 Pa applied to the underside of the sieve to remove and transport particles finer than the screen into a collecting jar. The weight of the overs or remains on the screen were transferred to the next largest sieve and the process was repeated with progressively higher screen sizes until all material passed through. Sieves with 32, 53, 75, 106, 125, 150, 180, 212, 250 and 300 microns were used.

#### 2.4.2. Water Absorption Capacity and Oil Absorption Capacity

Water absorption capacity (WAC) was measured as in a previous study described, but with modification [19]. Samples of 2.5 g were placed in centrifuge tubes with 30 mL of deionized water. To disperse the sample, the slurries were vortexed for 30 s. Samples were then allowed to sit at room temperature for 30 min with 2 additional agitations in that time. Samples were centrifuged at 3000× *g* for 30 min and the water was carefully decanted. WAC was calculated using the following equation:

(3) $$WAC(g\;water/g\;protein)=\frac{W_{f}-W_{i}}{W_{i}}$$

where W_f_ is the weight of the sediment and W_i_ is the initial weight of the sample.

Oil absorption capacity (OAC) was measured similarly, using the methods described with some modification [20]. Samples of 2.5 g were placed in centrifuge tubes with 30 mL of sunflower oil. Samples were shaken until the sample was dispersed and allowed to sit at room temperature for 30 min. Samples were centrifuged at 3000× *g* for 30 min and the oil was carefully decanted. The tubes were then inverted, allowing excess oil to drain for 20 min. OAC was then calculated using the following equation:

(4) $$OAC\left(g\;oil/g\;protein\right)=\frac{W_{f}-W_{i}}{Wi}$$

where W_f_ is the weight of the sediment and W_i_ is the initial weight of the sample. WAC and OAC were measured in triplicate for each sample.

#### 2.4.3. Least Gelation Concentration

The least gelation concentration (LGC) of each treatment was obtained by dispersing different concentrations of pea and soy proteins (12–20% *w*/*v*) in 10 mL of DI water in 1 cm diameter test tubes. The solutions were then heated, uncovered, at 95–100 °C for 1 h, immediately cooled via a cold-water bath, and then kept at 4 °C for 2 h. Wheat proteins were not tested since they are hydrophobic in nature and clump upon the addition of water. The LGC was determined, after chilling, as the concentration that forms a stable gel that does not drop or run when the test tube is inverted.

#### 2.4.4. Rapid Visco Analyzer Viscosity

A rapid visco analyzer (RVA) (RVA 4500, Perten Instruments, Waltham, MA, USA) was used to measure the pasting properties of each treatment using the AACC Method 76–21.02, as employed previously for flours [21]. Protein slurries at 15% solid concentration (*w*/*v*) were manually mixed so that protein clumps were better dispersed and there was reduced noise in the results plot. Slurries were placed in the RVA within 1 min of the initial mixing. Slurries were heated to 50 °C and held for 1 min, with initial stirring at 960 rpm for 10 s. For the remainder of the test, slurries were stirred at 160 rpm. Slurries were then heated to 95 °C at 12 °C/min, held for 2.5 min and then cooled again to 50 °C. Peak viscosity, time and temperature of peak viscosity, and end viscosity were measured and recorded. All RVA tests were conducted in triplicate.

#### 2.4.5. Differential Scanning Calorimetry

Protein denaturation, as shown by enthalpy, was measured via differential scanning calorimetry (DSC) with a Q100 V9.9 Build 303 (TA Instruments, New Castle, DE, USA) and analyzed with the Universal Analysis Program, V4.5A (TA Instruments). Comparing the DSC results of raw commercial protein isolates can be helpful to understand the impact of isolation processing on denaturation. DSC was conducted according to Brishti et al. (2017) with a few modifications [20]. Raw samples of 8–10 mg dry matter were weighed into stainless steel, high volume, hermetically sealed pans. Samples were equilibrated to 20 °C and were heated to 250 °C at a rate of 10 °C/min. An empty pan served as a reference. The nitrogen purge flow was 50.0 mL/min. The start and peak denaturation temperature and enthalpy of denaturation were recorded. Tests were conducted in triplicate.

#### 2.4.6. Molecular Weight

The molecular weight of each legume protein (raw and extruded) was qualitatively understood through sodium dodecyl sulphate polyacrylamide gel electrophoresis (SDS-PAGE) in non-reducing conditions. Extruded proteins were ground and sieved to less than 500 µm. Protein was extracted for an hour with deionized water (15 μg:1 mL) and then centrifuged for 5 min at 8000× *g*. The supernatant was then mixed with Laemmli buffer (2 supernatant: 1 buffer) and heated for 10 min in a boiling water bath. The 4x Laemmli sample buffer contained 277.8 mM Tris-HCl (pH 6.8), 44.4% (*v*/*v*) glycerol, 4.4% LDS and 0.02% bromophenol blue (Bio-Rad Laboratories, Inc, Hercules, CA, USA).

The prepared sample (12 μL) was pipetted into the gel lanes. Precision Plus Protein Standard (Bio Rad Laboratories, Hercules, CA, USA) was added at 5 μL and contained protein markers from 10–250 kDa. Electrophoresis was then conducted at 200 V, 25 mA, and 250 W to separate the proteins by molecular weight with 12% separating gel and 4% stacking gel. After electrophoresis, samples were fixed and stained using Brilliant Blue R concentrate. Samples were then destained overnight with 10% acetic acid, and further destained with deionized water.

#### 2.4.7. Phase Transition Analysis

Phase transition analysis (PTA) was used to measure the raw material softening and flow point temperatures. The test was conducted on a Phase Transition Analyzer (Wenger Manufacturing) with samples hydrated to 24% [22]. Raw treatments (2 g) were compressed in the chamber with a blank die to 120 bars for 15 s. A pressure of 100 bars was applied as the sample was heated at a rate of 8 °C/min, with a starting temperature between 5–7 °C. After the softening point of the material was measured, a 2 mm capillary die was placed under the sample and compressed again to 120 bars for 15 s, with 100 bars of pressure thereafter. Wheat gluten treatments required using lower pressure; 75 bars of consistent pressure were used throughout the test. When material began to flow through the capillary die, the compressing rod displacement changed, showing the flow point, and the temperature was marked as the flow temperature.

Extruded material was also tested to determine changes in flow point temperature compared to the raw material. For this analysis, raw materials were extruded on a lab-scale, Micro-18 extruder (Micro-18, American Leistritz, Somerville, NJ, USA) in order to impart moderate shear energy for the initiation of protein crosslinking but not to the extent of macromolecular degradation typically observed in high-energy pilot-scale extrusion. A comparison of PTA flow temperature before and after moderate shear transformations can help understand the potential for protein networking prior to pilot scale extrusion. Raw materials were hydrated to 24% MC before extrusion and run at a 3.3 kg/h throughput and a screw speed at 550 RPM. The material was extruded through the barrel sections with temperatures of 30, 40, 55, 95, 120 and 140 °C. An oval die with width of 5.5 mm and length of 3.0 mm was used to make ropes of extrudate. The extrudate was not dried, was ground finer than 250 microns with a Wiley mill, hydrated to 24% moisture, and run on the PTA using the parameters described above. Both raw and extruded material PTA tests were conducted in triplicate.

### 2.5. Extrudate Analysis

#### 2.5.1. Product Structure

The bulk density of extrudates was measured by measuring the mass of dried product filling a one-liter volume cup. The product’s internal structure was captured via pictures of longitudinal (along the direction of extrusion) and of transverse or horizontal (perpendicular to the direction of extrusion) sections. Measurements were completed in duplicate.

#### 2.5.2. Water Holding Capacity

Water holding capacity (WHC) is the measurement of water that is held within the structure of the final product, measured according to Kearns, Rokey and Huber (1989), with modifications [23]. Milled samples (15 g) were soaked in excess, room temperature water for 20 min, and then drained on a mesh screen for 5 min. Tests were conducted in triplicate. WHC was calculated using the following equation:

(5) $$WHC\left(\%\right)=\frac{Final\;weight-Initial\;weight}{Initial\;weight}\times 100$$

#### 2.5.3. Textural Analysis

Hardness, springiness and chewiness characteristics were measured using a TA-XT2 Texture Analyzer (Texture Technologies Corp., Scarsdale, NY, USA), programmed for a two-cycle texture profile analysis (TPA) compression test [22]. Analysis was performed on ground and rehydrated product to understand the textural qualities of the uniform extrudate without any binders, and also on patties formed using binders. For the former, treatments were rehydrated to 60% moisture. A back-extrusion cup was utilized to contain 20 g of the sample that filled, approximately, up to 1 cm in height in the cup, depending on the product density. The two-cycle compression test involved compressing up to 70% of the total distance with a circular aluminum probe. Textural properties were measured for patties formed in accordance with guidelines from the American Meat Science Association [24]. Patties were made with 91.5 g of each treatment and pressed to 1 cm thickness. The formula for plant-based patties is shown in Table 5. Patties were pan-broiled with no oil until an internal temperature of 71 °C was reached. Patties were allowed to cool to room temperature and a 2.5 cm core was taken from the center of 10 patties. The two-cycle TPA compression test was conducted on the cores at room temperature, compressing them to 70% of the total distance with a circular aluminum probe. Patties based on ground chicken, beef and pork and a commercial plant-based meat product (Beyond Beef^®^, El Segundo, CA, USA) were also tested using TPA as benchmarks and for comparison with the textural attributes of products obtained in this study. All tests were replicated 10 times.

### 2.6. Experimental Design and Statistical Analysis

A single factor experiment design structure was used with 8 treatments (or raw materials formulations), as described in Table 1 and Section 2.1. The independent variable was raw material formulation, while the dependent variables included raw material properties, processing characteristics such as SME and end-product attributes. The number of replicates is mentioned in the Methods sub-section corresponding to each test. All measurements were based on technical replicates, meaning that the same analytical procedure was applied to different samples, which, however, were not produced in replicate extrusion experiments. The extrusion treatments were not repeated, but for each treatment, different samples for any particular analysis were randomly selected from a group of extrudates weighing 10–16 kg and produced over a 15–20 min period. One-way ANOVA was performed to compare means and differences with SAS software (SAS, Cary, NC, USA). ANOVA was followed by Tukey’s test to determine the significance of differences and control for Type 1 errors (*p* < 0.05).

## 3. Results and Discussion

In this section, a careful scientific analysis is presented on the linkages between the chemistry and the physico-chemical properties of plant proteins, and in turn the impact on extrusion characteristics and degree of cross-linking due to processing. Finally, all of these data have been tied together with end-product quality such as porosity, layering, water holding capacity and texture.

### 3.1. Raw Material Characteristics

#### 3.1.1. Particle Size Distribution

**Figure 1:**
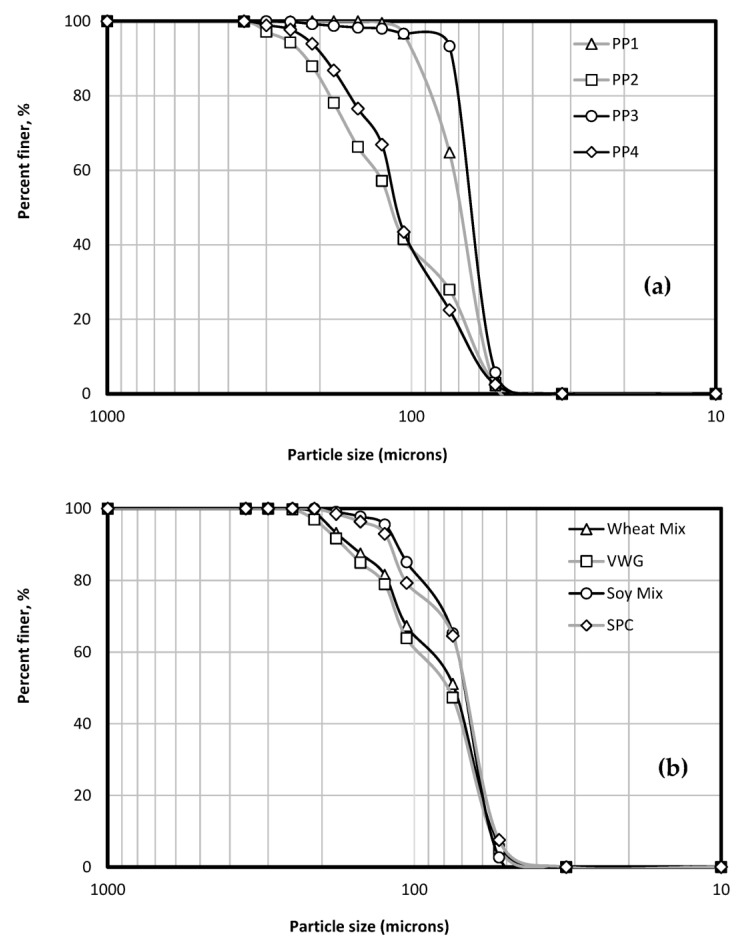
Particle size distribution of (**a**) pea protein and (**b**) wheat and soy treatments as the cumulative percentage of particles that passed through each sieve.

Particle size can vary based on industrial processing due to varying temperatures, vaporization, and air–water interface which can cause the increased denaturation and aggregation of hydrophobic regions [14]. PP1 and PP3 had a significant portion of their particles under 75 microns, as did the soy treatments (Figure 1). PP2 and PP4, however, had a wider particle size distribution with substantial portions ranging from 75 to 180 microns. VWG had 47% under 75 microns, but the remaining portion (about 55%) was spread all the way to 250 microns. Thus, PP1 and PP3 had the most uniform particle size for pea treatments, while SPC and the soy mix also had a relatively uniform particle size.

#### 3.1.2. Water Absorption Capacity and Oil Absorption Capacity

**Figure 2:**
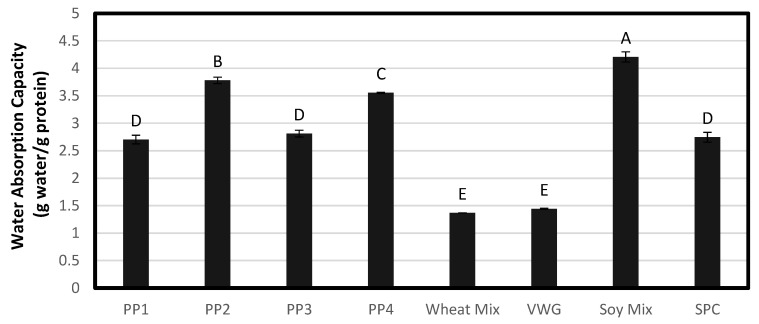
Raw material average water absorption capacity for all treatments. Bars denoted by the same letter are not significantly different (*p* < 0.05).

**Figure 3:**
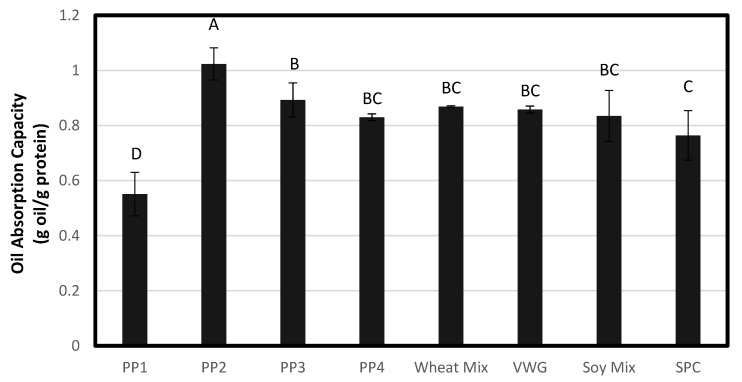
Raw material average oil absorption capacity for all treatments. Bars denoted by the same letter are not significantly different (*p* < 0.05).

The WAC and OAC of each treatment can be seen in Figure 2 and Figure 3. The WAC and OAC of each of the pea treatments is within the same range as previously reported [25]. The differences between the pea proteins in their ability to absorb water at room temperature may be partially due to the hydrophilic versus hydrophobic nature of the proteins, which in turn depends on the protein sub-units, and also their structure, and any transformations occurring during the commercial production process. VWG and Wheat Mix displayed a relatively low WAC (1.4) due to the hydrophobic nature of wheat gluten. Unlike leguminous proteins such as those found in soy and yellow peas, which have albumins or globulins as the major protein fraction, wheat gluten contains prolamins and glutelins that are soluble in alcohol and acid, respectively, rather than water or salt solutions [26]. The way gluten interacts with water is therefore quite different, and results in the low WAC observed. Soy Mix had the highest WAC of 4.2 as it comprised 50% soy protein isolate, which is typically highly water soluble. SPC had a moderate WAC (2.8), while the four pea protein isolates had a moderate to high WAC (2.7–3.8). Among the pea proteins, PP2 and PP4 had the highest WAC (3.8 and 3.6, respectively), which indicated a relatively high water solubility like soy protein isolate. On the other hand, PP1 and PP3 had a moderate WAC (2.7 and 2.8, respectively), pointing to a similar hydrophilicity and functionality to soy protein concentrate. This contrast between the four pea proteins is most probably due to differences in the isolation process during production, as they were obtained from four different commercial sources. The role of protein hydrophilicity versus hydrophobicity in the texturization process will be discussed in a later section.

PP2 had the highest OAC. Most proteins exhibited a similar OAC, but PP1 had a substantially lower OAC. Though gluten is a hydrophobic protein, it did not exhibit a higher OAC. Overall, the OAC of these proteins may be due to their physical attributes such as particle size, space between particles and agglomeration, rather than the affinity of the ingredients. Because PP2 had a greater particle size, it would be more agglomerated, with greater interstitial spaces and not as compact, and thus would be able to hold more liquid between the particles, while the small-particle-size PP1 would pack well and not hold much oil. PP2 and PP4 had a similar particle size distribution, but the higher water affinity of the former, as indicated by greater moisture content and WAC, caused more agglomeration of the particles, allowing a greater retention of oil.

#### 3.1.3. Least Gelation Concentration

LGC is a test used to determine the heat-gelling properties of proteins [27,28]. Heat gelation is the ability of a protein to form a three-dimensional network through its denaturation and aggregation [29]. The structure is held by protein–protein interactions, bonds, and electrostatic forces. In a gel, the protein also interfaces with a solvent (water, in the case of the LGC test) held within the network [28]. LGC tests specifically for thermogelation, the same mechanism which occurs during extrusion, by determining the concentration at which a protein can form a gel in water after heating. Thus, unlike WAC, which is a cold-water solubility test, LGC is hot-water solubilization process. Measuring gelation properties is helpful as it can help to further characterize various proteins, and indirectly point to differences in their chemistry, structure and functionality. Moreover, the gelation of proteins under heat, pressure and shear is what helps create and solidify the fibrous and layered structure of meat analogs during extrusion [30].

Most PPIs had a relatively moderate heat-gelling ability, requiring at least 16% solids to gel (Table 6). The exception was PP1, which had a high heat-gelling ability or low LGC (14%). SPC also had a low LGC of 14%. Interestingly, Soy Mix had the least heat-gelling ability and required the highest solids concentration (18%) to create a firm gel, even though its protein content was similar to the pea proteins and higher than SPC. Thus, LGC was not a function of the content of protein, but more related to its chemistry and structure. For example, in a previous study, stronger gels came from pea proteins that were less fractionated [12].

It is also important to note that the protein isolation process manipulates the protein structure and processes vary throughout the industry [31]. Thus, proteins could be exposed to treatments that would allow for various gelation behaviors. The gelation concentration of globular proteins is affected by a number of factors, but especially by the pH and ionic strength the protein is exposed to, as well as enzymes and pressure treatment [32,33]. It is by various combinations of these treatments, too, that different proteins may test at the same LGC but for different molecular-level reasons. With the amount of factors that change protein properties, comparison between protein sources is difficult [33]. Higher solubility can be achieved through hydrolysis but results in a tradeoff of lower gel strength [33]. This could be an indication that some of the pea proteins may have been processed in a way to increase solubility at the expense of gel strength, thus leading to a higher critical concentration of protein. Gelation properties as determined using the LGC test were further confirmed using RVA pasting properties, which is a rheological test discussed in the next section.

Despite the same LGC of PP1 and SPC, extrusion outcomes varied greatly in terms of internal structure and density (64 g/L and 253 g/L, respectively). Even within the same type of protein and with the same LGC, the final structure and density varied for PP2, PP3, and PP4 (65–172 g/L). LGC is a measure of gelation properties in a very dilute dispersion (12–20% solids), whereas protein network formation during extrusion-based texturization occurs in a much dryer processing environment (60–70% solids). Therefore, obvious differences exist between the two processes, including stronger protein interactions in the latter. Thus, LGC is not the sole determinant of product structure or extrusion characteristics, even though it is helpful in understanding differences between proteins. The role of heat gelation ability and LGC in the texturization process is discussed in more detail in a later section.

#### 3.1.4. Rapid Visco Analyzer Viscosity

**Figure 4:**
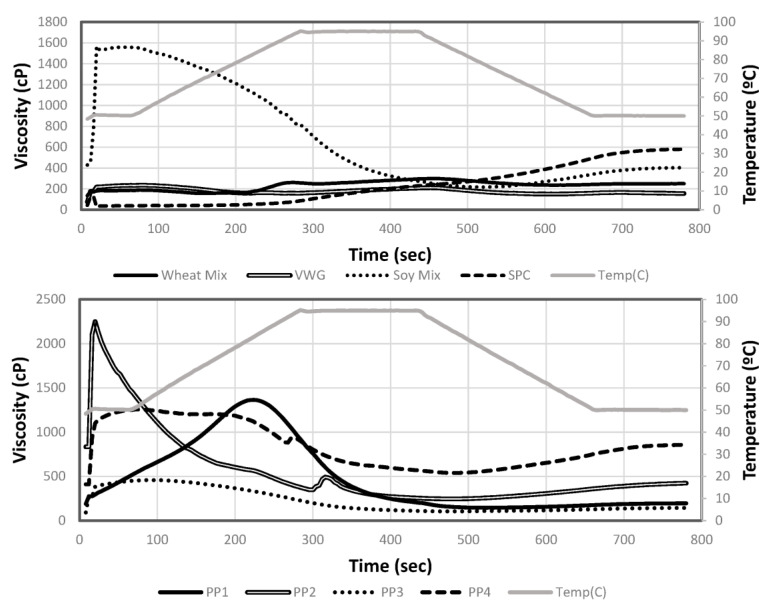
Rapid visco anlayzer (RVA) average viscosity curves for wheat and soy proteins (**top**) and pea proteins (**bottom**) using 15% solids concentration of raw materials.

Pea protein solutions displayed different behavior upon hydration, heating and low shear RVA testing. Average RVA curves are shown in Figure 4 and corresponding pasting property data are summarized in Table 7. Among pea proteins, only PP1 had an increase in viscosity during heating, with a peak viscosity of 1387 cP at 83 °C. PP1 was also the pea protein treatment with the highest heat gelation ability, having the lowest LGC that almost matched the concentration at which RVA tests were conducted (15%). Thus, both RVA and LGC data point to the same thermally induced gelation and swelling properties of PP1, where the heat allowed proteins to solubilize in water and increase the viscosity. All other pea proteins had peak viscosities prior to the commencement of heating (51–55 °C), demonstrating moderate to high cold-water swelling properties, unlike PP1. The pea protein PP2 had a very high peak viscosity of 2250 cP at the outset of testing and before heating, thus showing that it had instant hydration and swelling properties. PP4 also demonstrated quick swelling but a relatively lower peak viscosity (1257 cP) before heating. Both PP2 and PP4 also had the highest WAC among the pea proteins, as discussed earlier. On the other hand, PP3 demonstrated moderate cold-water swelling with a low peak viscosity of 460 cP. Both the heat-swelling PP1 and moderately cold-swelling PP3 had the lowest WAC among the pea proteins. Correlation analysis found a relationship between WAC and the peak viscosity (0.7/*p* < 0.0001), although it was moderate, possibly due to the low WAC of heat-swelling proteins. Similar findings have previously related the WAC and viscosity of pea proteins, which found that a pea protein that was able to absorb more water resulted in a higher viscosity [25].

Similarly, among the non-pea proteins, Soy Mix demonstrated instant hydration and cold-swelling properties with a peak viscosity of 1626 cP observed at 56 °C. Soy Mix also had the highest WAC among all proteins. This cold-water solubility was due to the soy protein isolate component. VWG and Wheat Mix exhibited no cold-water swelling during RVA testing and only a slight heat swelling. These two also had the lowest WAC among all the proteins. This reflects the hydrophobic nature of wheat gluten as discussed earlier. SPC exhibited heat-swelling properties, with RVA viscosity increasing steadily to 320 cP while the temperature remained higher than 70 °C, and further increasing to a final viscosity of 580 cP on cooling to 50 °C. The heat-induced solubilization and gelation properties of SPC could also be inferred from its low LGC as discussed earlier.

It is clear that RVA testing represents a combination of WAC and LGC, which are the cold-water solubility and heat gelation tests, respectively. Thus, a rheological test such as RVA in combination with WAC and LGC can be used as a useful set of physico-chemical analyses to characterize proteins by their cold-swelling and heat-swelling categories. PP2 and Soy Mix can be categorized as having high cold-water swelling properties, PP3 and PP4 as moderate cold-water swelling, and PP1, SPC, VWG and Wheat Mix as low cold-water swelling. On the other hand, PP1 and SPC can be categorized as having heat-swelling and -gelling properties. The relationship between these characteristics and texturization properties on extrusion are described in the extrudate analysis section.

It should be noted that the impact of starch, fiber and hydrocolloids on the pasting and gelling properties of raw materials can be significant, but the overall carbohydrate content is 10% or less for each ingredient investigated in this study, as can be seen from Table 2. Thus, the impact of these components is minimal as compared to the proteins that are at the level of 80% or more. Thus, it is the proteins that primarily controlled the cold- and heat-swelling properties of the various materials in this study.

#### 3.1.5. Differential Scanning Calorimetry

**Figure 5:**
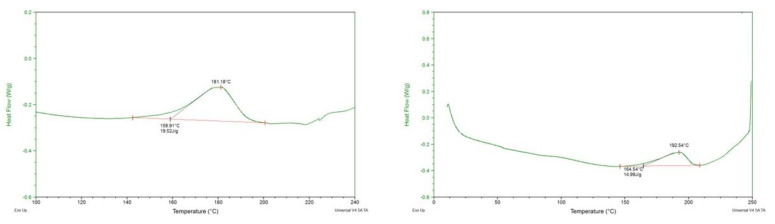
DSC thermograms showing start and peak temperature for protein denaturation and enthalpy for denaturation for two representative ingredients—PP1 (**left**) and soy mix (**right**).

**Figure 6:**
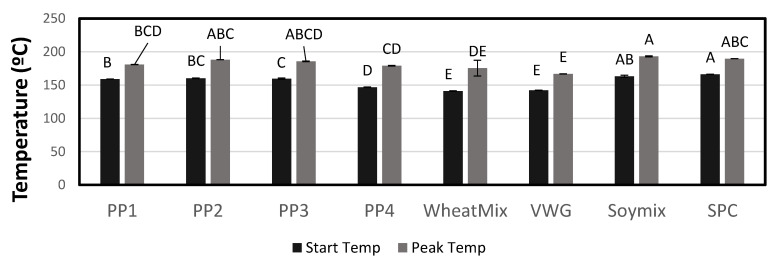
Mean starting and peak temperatures of denaturation for protein in each treatment as determined by DSC. Bars denoted by the same letter are not significantly different (*p* < 0.05).

**Figure 7:**
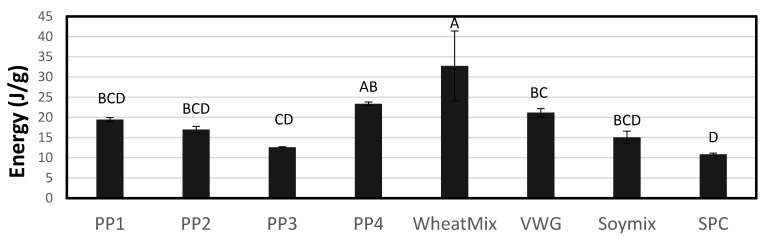
Mean energy required to denature proteins in the raw material as determined by DSC. Bars denoted by the same letter are not significantly different (*p* < 0.05).

DSC thermograms showing start and peak temperature for the denaturation and enthalpy of denaturation for two representative ingredients are shown in Figure 5. No other thermal event was identified in any of the protein samples except for protein denaturation, as can be seen from the representative thermograms. The DSC thermograms of other samples were similar but had different values for these denaturation characteristics, as described below. The starting and peak temperature of denaturation were different among the pea proteins, ranging from 146–160 °C and 179–188 °C, respectively (Figure 6). Wheat treatments were lower than pea proteins in starting and peak temperatures of denaturation, while soy was higher. The enthalpy measured during denaturation varied as well (Figure 7). PP3 required the least amount of energy (12.6 J/g), while PP4 required the most (23.4 J/g). PP1 and PP2 required 19.4 and 17.0 J/g, respectively. The soy treatments required less energy to denature (10.9–15.1 J/g), while the wheat treatments required the highest (21.2–32.7 J/g). The heat denaturation properties of proteins are not clearly related to their cold- and hot-water swelling characteristics described earlier. Among pea proteins, PP1 required one of the highest enthalpies to denature, meaning it would require heat to denature, form a gel and build viscosity, as seen with RVA testing. PP4, though it also required a high enthalpy, had a much higher WAC, which led to a higher initial viscosity and thus no further increase in viscosity during heating.

These DSC results are not indicative of the properties of native proteins or their unfolded structures, as varying levels of denaturation might have occurred during the isolation and commercial production process of the different proteins. A previous study found that lower denaturation enthalpies are a result of harsher or longer thermal treatments [15]. Greater protein denaturation during isolation might explain the low enthalpy for PP3, due to the high prior unfolding of the protein structure, while PP4 may have the least denatured protein. This is, however, speculative, as information on the processing and isolation of proteins was not available. The relatively high enthalpies of VWG and Wheat Mix might be indicative of the relatively less aggressive wet milling process used in the isolation of wheat gluten.

#### 3.1.6. Molecular Weight Analysis (SDS-PAGE)

Proteins differ based on their cultivar and extraction methods [14,34]. Having a molecular level understanding of proteins can be useful in understanding their gelation upon heating, and for pea proteins, this generally means understanding the solubility and the content of legumin, vicilin and convicilin. Legumin is attributed to disulfide bonding during gelation and texturization, due to the greater presence of sulfur-containing amino acids, but the gelation functionality of legumin can vary by variety [35]. Vicilin lacks the sulfur content of legumin, yet the convicilin subunit of vicilin still contributes to gelling. The core of convicilin is largely the same as vicilin, but convicilin is distinguished from vicilin because of a highly charged end of the protein which allows it to form the gel network [35].

**Figure 8:**
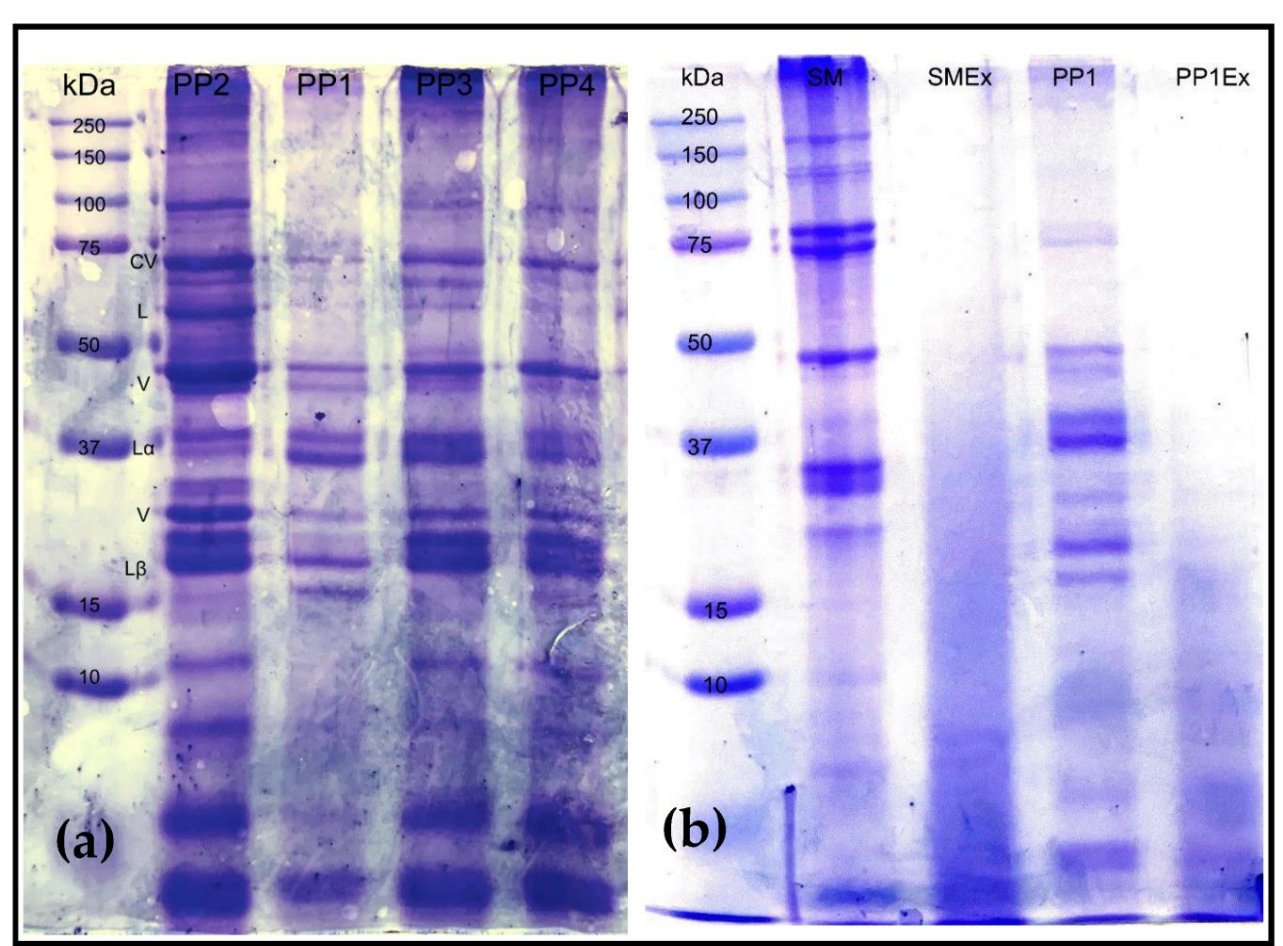
Non-reducing SDS-PAGE gel for raw pea proteins (**a**) and select raw and extruded proteins (**b**). Columns in (**a**) are the standard marker, PP2, PP1, PP3 and PP4. Note that PP2 comes before PP1. Columns in (**b**) are the standard marker, raw soy mix (SM), soy mix extruded (SMEx), PP1 and PP1 extruded (PP1Ex). CV, Convicilin; L, Legumin; V, Vicilin; Lα, acid subunit of legumin; Lβ, basic unit of legumin.

Due to the lower intensity SDS-PAGE bands of unextruded PP1, this protein seems to be less soluble in water than the other proteins (Figure 8a). This observation is compatible with the lack of cold-water solubility of PP1 and its heat-swelling nature observed in the WAC, LGC and RVA tests. PP2 is the most soluble, as noted by the greatest intensity of the bands. The high solubility of PP2 gives an explanation of its high WAC and the RVA cold-swelling viscosity. After immediate hydration, PP2 is able to take in water and create viscosity, but upon heating and mild shear, the protein network begins to disintegrate and viscosity decreases. Unextruded PP3 and PP4 also exhibit relatively dark SDS-PAGE bands, although of less intensity than PP2, reflecting their low-to-moderate cold-swelling properties described earlier. Bands associated with a molecular weight higher than 70 kDa are present in PP2 but absent in PP3 and PP4. This could be the reason for the lower cold swelling and lower RVA peak viscosities observed in the latter. The unextruded Soy Mix had relatively high intensity bands at 70 kDa and above (Figure 8b), although distinct from PP2, which is consistent with its higher cold-swelling properties due to the soy protein isolate.

As can be further seen from Figure 8a, the more cold-soluble proteins (PP2-PP4) have 70 kDa bands of more intensity, indicating a higher presence of convicilin, and each of these had a slightly higher LGC, which could be due to the electrostatic repulsion preventing gelling and requiring slightly more protein to network. With a higher convicilin content, more N-terminus negative charges exist and therefore more repulsion occurs, requiring more protein to make a gel [30].

SDS-PAGE of select extrudates showed obvious change in the molecular weight and solubility of the proteins compared to that prior to extrusion (Figure 8b). No distinct bands were present after extrusion. During low-moisture extrusion, previous studies found that the vicilin protein was unaltered while legumin changed after extrusion, likely via aggregation and an increase in molecular weight [25,36]. Extrusion texturization is a continuous thermomechanical process that transforms globular proteins such as pea or soy proteins, or irregular plant proteins, such as wheat gluten, into meat-like fibrous structures. During this process, the moisturized protein matrix undergoes several physical, chemical and structural changes that significantly affect the textural quality of the extruded products. To form microscopic and macroscopic fibers, the proteins need to unfold, cross-link, and align themselves. Covalent bonds, such as peptide and disulfide bonds, and non-covalent interactions, including hydrogen bonding, hydrophobic interactions and ionic linkage, undergo alterations, and new bonding is formed through physical and chemical cross-linking. These mechanisms led to the proteins in this study becoming aggregated and insoluble after extrusion, rendering a gel with no distinct bands.

#### 3.1.7. Phase Transition Analysis

**Figure 9:**
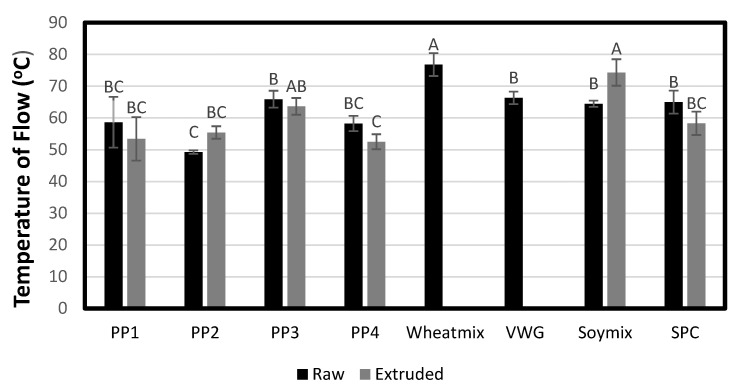
Flow temperature for raw material treatments and moderately sheared extrudate. Bars denoted by the same letter are not significantly different (*p* < 0.05). Wheat gluten was not able to be hydrated and extruded on the lab-scale extruder, thus no extruded PTA test was conducted on Wheat Mix and VWG.

**Figure 10:**
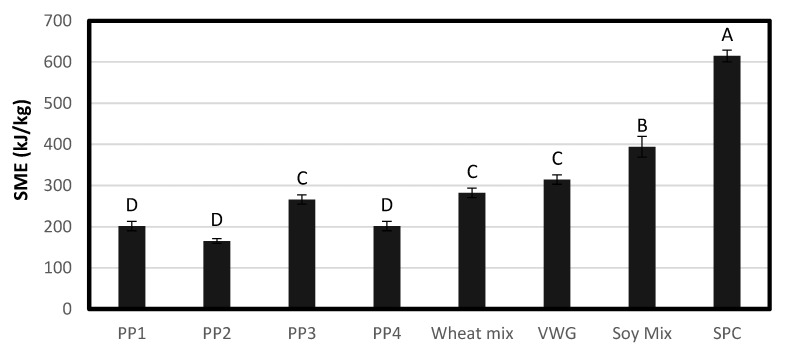
Mean SME required by each treatment during extrusion processing. Bars denoted by the same letter are not significantly different (*p* < 0.05).

The various raw materials showed different phase transition behaviors (Figure 9). The temperature at which the material began to flow through the PTA capillary die was highest for soy and wheat proteins (64.5–76.8 °C), indicating a high resistance to flow. Among the pea proteins, the flow temperature was highest for PP3 (65.9 °C) and lowest for PP2 (49.2 °C). With a higher temperature required to achieve a flowable melt, it follows that more energy will be required to process the material in the extruder. The thermal energy required for raw materials to flow in the PTA mirrored the specific mechanical energy (SME) required for pilot-scale processing (Figure 10), as is discussed in the following section.

The PTA flow temperature after moderate energy extrusion (using a lab-scale system) is also shown in Figure 9. The change in flow temperature between raw and extruded proteins can be a useful bench-top analysis tool for determining their cross-linking potential, as was described in a previous study by our research group [22]. In the current study, PP2 and Soy Mix were the two proteins that showed an increase in flow temperature (from 49.2 to 55.4 °C and 64.5 to 74.3 °C, respectively). This indicated an increase in resistance to flow or viscosity due to high degree of protein networking or cross-linking induced by heat, shear and pressure during moderate extrusion. It is interesting to note that PP2 and Soy Mix also exhibited cold-water swelling properties as described earlier. Thus, cold-swelling proteins might intrinsically have a higher cross-linking potential. The relationship between protein cross-linking or networking potential is thus determined and product texturization and final quality is discussed in the extrudate analysis section. The protein networking induced in the other proteins due to moderate extrusion might be of a lesser degree and possibly dwarfed by any macromolecular degradation during the extrusion and grinding process, as can be seen from the slight reduction in flow temperature.

#### 3.1.8. Extrusion Processing

Among pea proteins, SME was greatest for PP3 (266 kJ/kg), while PP2 had the lowest SME (165 kJ/kg) as can be seen from Figure 10. The highest SME was required for the processing of wheat and soy treatments (282–615 kJ/kg). These trends in general were similar to that of the raw material PTA flow temperature. Thus, the latter appeared to be a good indicator of extrusion SME, as has been shown previously as well [37]. Both PTA flow temperature and extrusion SME measurements are an aspect of the resistance to flow of raw materials. A raw material with a higher flow temperature requires more energy to flow, which means that the material will have greater resistance to flow during extrusion and require more SME for processing.

SME might also have some relation to the swelling or water hydration properties, as well as particle size and functionality of the proteins. For pea proteins, the SME has an inverse trend to their WACs. The more water the protein is able to hold, the less energy it requires to process. During extrusion, each pea protein was processed with the same amount of water. A reason for the low SME of PP2 (165 kg/kJ) may be the high solubility the protein has, which means it will not build viscosity as well, and it would require less energy to process. To attain the same SME with a more soluble protein such as PP2, a lower in-barrel moisture or IBM may be required, so that the melt can have greater viscosity. PP2 and PP4 had the largest particle size and the lowest SME of the pea protein treatments. In at least one previous study involving corn meal extrusion, a larger particle size was observed to have a lower SME due to easier flow [38]. SPC had the most water added to it during extrusion, which would generally plasticize the melt and reduce the viscosity and SME in the extruder. Still, the greatest SME was found in SPC (615 kg/kJ). This may be due to the presence of more functional, heat-swelling and viscosity-building proteins. Indeed, SPC has a higher heat-induced viscosity than most pea proteins and wheat treatments, as was observed in RVA data.

### 3.2. Extrudate Analysis

#### 3.2.1. Visual Analysis

**Figure 11:**
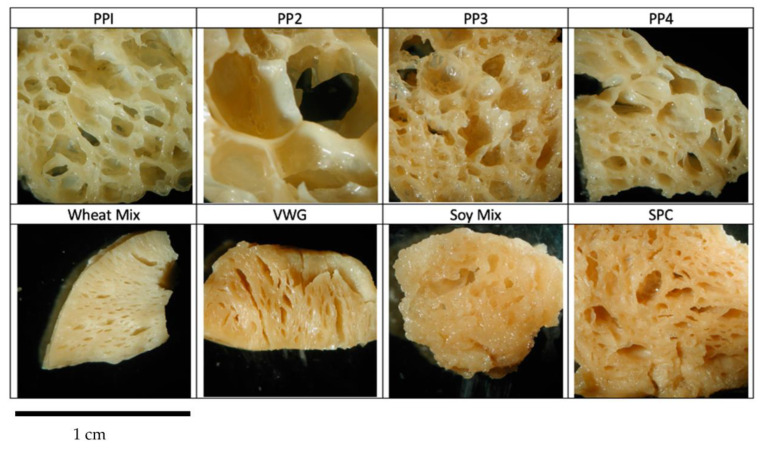
Horizontal cross sections of whole extrudate pieces. Horizontal cross sections are cut against the direction of flow from the extruder. Each image is approximately 1 cm across.

**Figure 12:**
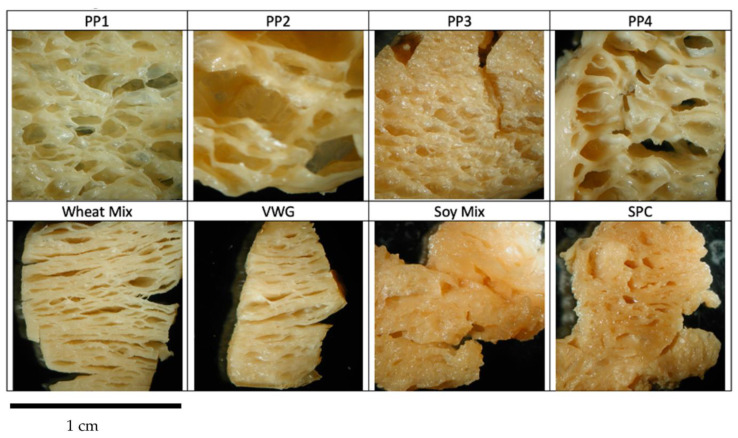
Longitudinal cross sections of whole extrudate pieces. Longitudinal pieces were cut along the direction of flow from the extruder. Each image is approximately 1 cm across.

Pictures of product transverse (perpendicular to extrusion direction) and longitudinal (along extrusion direction) cross sections are shown in Figure 11 and Figure 12. All extrudates were clearly texturized, as evident from their cohesive internal structure; however, they differ substantially in porosity and extent of layering. In general, a relatively porous structure was observed due to the product expansion that is typical for the low-moisture and higher-energy texturization process used in this study. Among pea proteins, the product based on PP2 displayed a very cellular, porous structure, followed by PP4 and PP3, with PP1 showing the least expanded internal structure. The longitudinal cross sections show some layering or lamination along the direction of extrusion, more prominently in PP1 and PP3. The porous structure of pea-protein-based products may be attributed to the strong protein–protein networking induced during extrusion, which led to ‘film formation’ and expansion. This high degree of extrusion-induced cross-linking is typical of pea proteins [22]. This was particularly a feature of PP2, which also showed a higher cross-linking potential in PTA analyses. It is inferred that the cold-swelling nature of PP2 promotes cross-linking to the extent that the product expands into a cellular structure after texturization rather than forming dense layers. It is expected that if a moderate quantity (10–20%) of starch or fiber is present to inhibit the protein cross-linking, a more fibrous or layered texture would result, as found in previous studies by our group [22,39]. Conversely, the low cold-swelling nature of PP1 (combined with heat swelling) and PP3 led to better layering and a denser product structure. Similarly, in non-pea proteins, the internal structures of VWG and Wheat Mix exhibited the least porosity, a fibrous structure with dense layering in both the horizontal and longitudinal cross sections. This is consistent with the very low cold-swelling properties and hydrophobic nature of wheat gluten. Moreover, gluten is naturally a fibrous-shaped protein, while both pea and soy are globular proteins [30]. Thus, wheat gluten texturizes into a fibrous structure more easily than the other proteins. Although not clearly visible from the pictures, the Soy Mix product had a more porous structure, while SPC was denser and layered. This is also consistent with the high cold-swelling properties of Soy Mix and particularly its soy protein isolate component, and the heat-swelling and low cold-swelling properties of SPC. These observations on visual structure are, however, not substantiated by objective measurements of features such as layers or cells.

To summarize, all proteins were texturized or cross-linked in the pilot-scale extrusion process. However, the high cold-swelling proteins (PP2 and Soy Mix) exhibited a greater degree of cross-linking, as was observed visually with a more cellular or porous structure, and confirmed by benchtop analyses using PTA data. Heat-swelling and/or low cold-swelling proteins (PP1, PP3, VWG, Wheat Mix and SPC) exhibited a lesser, yet optimum degree of cross-linking, leading to a denser, layered and fibrous structure. Moderate cold-swelling proteins (PP4) had a structure somewhere in between the two.

#### 3.2.2. Bulk Density

**Figure 13:**
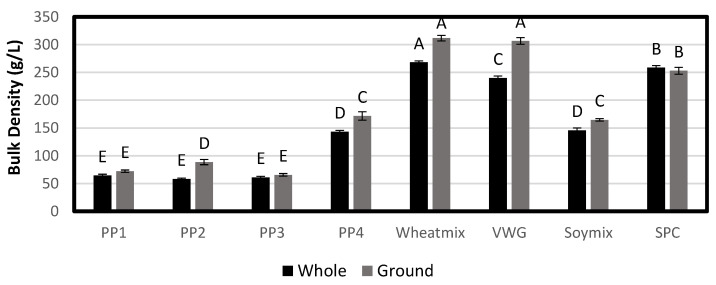
Average bulk density of whole extrudate and ground extrudate. Bars denoted by the same letter are not significantly different (*p* < 0.05).

The bulk density of pea protein extrudates ranged from 58 to 143 g/L, and was generally lower than that of non-pea extrudates (146–268 g/L) (Figure 13). This corresponded well with the higher porosity observed visually, as bulk density is inversely proportional to the degree of expansion. PP2 product had the lowest bulk density of 58 g/L, which was consistent with its highly porous, cellular internal structure and the high cold-swelling and cross-linking potential of the PP2 protein. Within non-pea treatments, the wheat treatments (240–268 g/L) and SPC (258 g/L) had the highest bulk density, which was consistent with their denser, layered structure and the heat-swelling and/or low cold-swelling nature of gluten and SPC proteins. On the other hand, Soy Mix had a relatively low bulk density of 146 g/L due to higher cross-linking and the cold-swelling nature of soy protein isolate.

In general, for the proteins studied, a higher SME resulted in a denser product. This was contrary to the usual observation for starch-based expanded extrudates, where higher mechanical energy input leads to a greater intensity of cooking and die temperatures and thus leads to more expansion and lower bulk densities [40]. The opposite trend in this study was due to the fact that the porosity of texturized protein extrudates was a function of the intensity of cross-linking, which in turn was more dependent on the nature and functionality of the protein than the energy input during extrusion.

#### 3.2.3. Water-Holding Capacity

**Figure 14:**
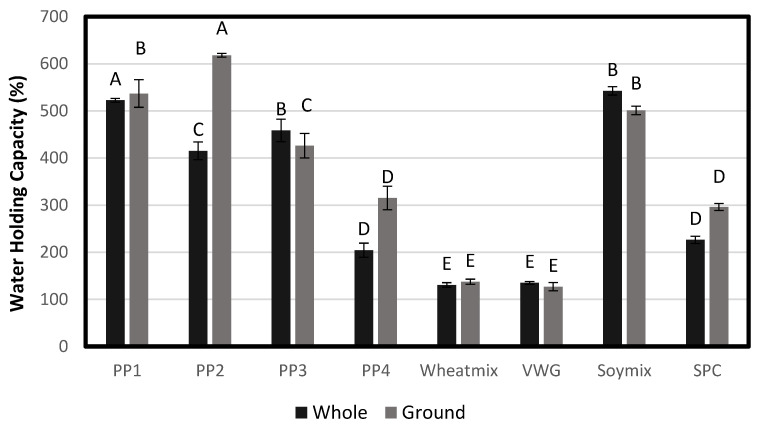
Water holding capacity (%) of whole extrudates and ground extrudates. Bars denoted by the same letter are not significantly different (*p* < 0.05).

Texturized vegetable protein products typically have a layered yet porous structure [4]. Previous work has shown that the internal structure of extrudates and the bulk density have a great impact on the extrudate’s WHC [22]. The open cell structure of some of the whole extrudates skewed the whole-product WHC trends, but ground-product WHC had a more consistent inverse relationship with bulk density, as has been observed previously (Figure 14). Extrudates with higher expansion and lower density resulted in higher ground WHC, as the micro-level porosity of the products was maintained even after grinding. For example, VWG, wheat mix and SPC had the lowest WHC (127–296%) and also the highest bulk density. These were also the products that exhibited relatively denser layering in the internal structure. A low WHC combined with high density can contribute to a lower sponginess of the hydrated product and a harder, more meat-like texture. On the other hand, pea protein treatments had a relatively high WHC (315–618%), which was consistent with their low bulk density. PP2 had the highest WHC of 618% and was also the most porous of all the products. Among non-pea treatments, Soy Mix had the highest WHC of 501% and also had the lowest density.

#### 3.2.4. Textural Analysis

**Figure 15:**
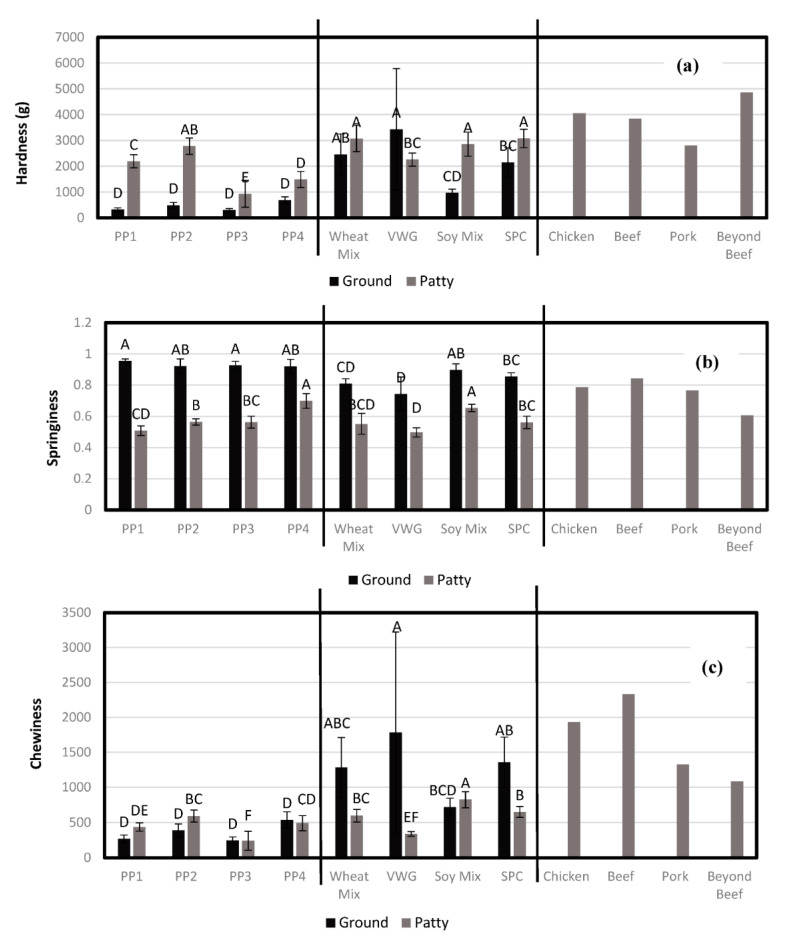
Mean hardness (**a**), springiness (**b**), and chewiness (**c**) of ground extrudate and patties made from the ground extrudate. Bars of the same color denoted by the same letter are not significantly different (*p* < 0.05).

The textural attributes of plant-based meat that mimics actual meat-like texture have not been extensively studied. Texture profile analysis (TPA) has been used to characterize plant-based meat, and in a very general sense, it was assumed that high hardness and chewiness and relatively low springiness could simulate meat muscle [22]. These primary TPA attributes for muscle-meat patties and plant-based meat products in this study are shown in Figure 15. The hardness of chicken, beef and pork patties ranged from 2804 to 4057 g (as compared to 4852 g for Beyond Beef^®^); springiness ranged from 0.77 to 0.84 (0.61 for Beyond Beef^®^); and chewiness from 1329 to 2333 (1083 for Beyond Beef^®^). Clearly chewiness was one aspect where the commercial plant-based patty was found lacking. This was due to low springiness but also probably due to low cohesiveness, which is another attribute used for calculating chewiness besides hardness.

Ground and hydrated pea-protein-based products (without binders) in this study did not compare well with the muscle meat or commercial plant-based meat (also based on pea protein) benchmarks, having very low hardness (304–692 g) and chewiness (246–538), and very high springiness (0.92–0.95). This was attributed to the high degree of cross-linking, porosity and WHC of these products. Similar results were found for whole hydrated pea-protein-based simulated meat products previously [22]. By adding binders (pea protein isolate, chickpea and methylcellulose), the corresponding patties showed an improvement in their textural attributes, with higher hardness (930–2772 g), similar or higher chewiness (242–594) and lower springiness (0.51–0.70). Most of these patty products still had too-low hardness and chewiness as compared to the benchmark products, except PP2, which interestingly, on the addition of binders, had the desired low springiness and also hardness similar to pork, which had the lowest value among all meats (2804 g). The chewiness of the PP2 patty was still much less than desired, but the use of a higher level of binders, or different binders, could possibly rectify that shortcoming.

Ground and hydrated non-pea-protein-based products (without binders) had much higher hardness (966–3433 g) and chewiness (721–1785), and lower springiness (0.74–0.90) than pea protein products, and compared reasonably well with chicken, pork and commercial plant-based meat benchmarks. The lowest hardness and chewiness and highest springiness was found for the Soy-Mix-based product, which was not surprising as that was associated with a higher level of cross-linking, porosity and WHC, as with pea protein products. As in the case of pea protein, the addition of binders in most cases led to an increase in hardness (2261–3078 g) and a decrease in springiness (0.50–0.65), but contrary to pea proteins, there was also a decrease in chewiness (340–825), which was not desirable for a meat-like texture. These products were nevertheless a better candidate for mimicking meat texture, and the use of different and better binders could potentially help in that regard to improve chewiness. Interestingly, Soy Mix treatment had the biggest improvement in hardness and even chewiness with the addition of binders, and its patty was closest to pork in texture. This, in combination with similar observations for PP2, pointed to the better compatibility of the set of binders used in the current study with these two cold-swelling proteins and the associated higher cross-linking, porosity and water-holding of the extruded products.

It should be noted that the TPA test for texture fails to account for mouthfeel, which can critically change the perception of the product. Sensory analysis to understand the mouthfeel differences in such products would be helpful, including attributes such as juiciness, cohesiveness of mass and surface properties (grainy, smooth, fibrous, lumpy, etc.), which can be expected to vary due to differences in porosity, layering and WHC. Though not many studies have been published on sensory analysis and the acceptance of plant-based meat products, in a study on high moisture extrusion (HME)-based soy and pea patties, sensory analysis data pointed to significant differences in the cohesiveness of mass, hardness and springiness of plant protein patties made with soy or pea protein, and a consumer study found that the overall texture of the soy patty, having greater hardness and springiness, was preferred to pea patties [41].

It should also be noted that hardness and chewiness data for ground VWG had high variation (standard deviation), since sample mass for all TPA tests were standardized to 20 g. As VVG samples had a very high bulk density compared to the other textured proteins, it resulted in a much thinner layer (low volume) sample as compared to other treatments. The lower thickness of the test samples led to inconsistent results and greater variability.

## 4. Conclusions

Pea proteins are innately different from wheat and soy proteins and thus resulted in different plant-based meat properties after low moisture extrusion. Formulations containing soy protein isolate and most pea proteins had highest cross-linking potential, required the least specific mechanical energy during extrusion and led to a porous and less layered, texturized internal structure. On the other hand, soy-protein-concentrate- and wheat-gluten-based treatments had a dense, layered extrudate structure. Protein characteristics including water absorption capacity, least gelation concentration, rapid visco analyzer pasting profiles and phase transition flow temperature provided helpful information for understanding the functionality, texturization via extrusion and internal structure of the product. In turn, structural attributes such as porosity and layering and the use of binders significantly impacted the texture of plant-based meat patties including hardness, springiness and chewiness. This knowledge can be applied to tailor formulations and also the extrusion process to particular proteins and the desired product quality.

## Figures and Tables

**Table 1:** Formulas (%) used in the extrusion treatments for plant-based meat.

Treatment	PP1	PP2	PP3	PP4	VWG	Wheat Mix	Soy Mix	SPC
Pea isolate 1	100							
Pea isolate 2		100						
Pea isolate 3			100					
Pea isolate 4				100				
Vital Wheat Gluten					100	90		
Wheat Flour						10		
Soy Protein Isolate							50	
Soy Protein Concentrate							50	100

**Table 2:** Composition of extrusion treatments as determined by proximate analysis and supplier specifications (%).

Component	PP1	PP2	PP3	PP4	VWG	Soy Mix
Protein	80.3	80.3	82.9	79.2	86.7	77.8
Carbohydrate	4.0	9.3	7.6	3.2	7.3	9.8
Fiber	4.0	0.2	1.0	2.7	0.2	1.8
Fat	6.0	0.5	0.4	6.0	0.9	0.6
Ash	1.6	4.1	5.3	4.1	0.4	4.6
Moisture	4.1	5.9	3.7	4.8	4.8	5.4
Total	100.0	100.0	100.0	100.0	100.0	100.0

**Table 3:** Extrusion parameters for each treatment. All parameters remained consistent for pea protein (PP) treatments, while optimization was required for Wheat Mix, vital wheat gluten (VWG), Soy Mix, and soy protein concentrate (SPC). The remaining variables were kept constant.

Extrusion Parameter	PP1	PP2	PP3	PP4	Wheat Mix	VWG	Soy Mix	SPC
Feed Rate (kg/h)	50	50	50	50	50	50	45	45
In-barrel moisture (%)	29.9	29.3	28.9	28.7	35.2	34.3	34.7	38.7
Screw Speed (rpm)	450	450	450	450	450	450	320	206
Venturi die size (in)	1/8	1/8	1/8	1/8	1/8	1/8	1/4	1/4

**Table 4:** Extruder screw configuration. The two screws differed only in the first two feeding elements.

Left	1	1	1	1	3	3B	1	1	4	4	5	6	5	6	5	5B	7
Right	2	2	1	1	3	3B	1	1	4	4	5	6	5	6	5	5B	7
1	Full pitch, double flight																
2	Full pitch, single flight																
3	Forward kneading block																
3B	Forward kneading block, backward																
4	¾ pitch, double flight																
5	Reverse kneading block																
5B	Reverse kneading block, backwards																
6	½ pitch, double flight, cut flight																
7	¾ pitch, double flight, cut flight, cone																

**Table 5:** Patty formulation for textural analysis. TVP refers to the extruded treatments as per Table 1.

Ingredient	Percentage
Textured Vegetable Protein (TVP)	59.25
Water	29.6
Pea Protein Isolate	1.5
Chickpea Flour	1.5
Sunflower oil	3
Methylcellulose	2.75
Salt	1
Beet Powder	0.75
Spices	0.65

**Table 6:** Least gelation concentrations for raw mixes based on pea and soy protein. The symbol ‘+’ means gel was observed the given concentration and the symbol ‘−‘ means gel was not observed. Gluten not tested due to its hydrophobicity.

Treatment	12%	14%	16%	18%	20%
PP1	−	+	+	+	+
PP2	−	−	+	+	+
PP3	−	−	+	+	+
PP4	−	−	+	+	+
Soymix	−	−	−	+	+
SPC	−	+	+	+	+

**Table 7:** Means and standard deviations of RVA-based rheological measurements. Means in a column followed by the same letter are not significantly different (*p* < 0.05).

Treatment	Peak Viscosity (cP)	Time of Peak Viscosity (s)	Temperature of Peak Viscosity (°C)	Final Viscosity (cP)
PP1	1387 ± 157 ^c^	223 ± 14 ^c^	83 ± 3 ^c^	195 ± 7 ^b^
PP2	2250 ± 52 ^a^	20 ± 0 ^e^	51 ± 0 ^e^	299 ± 3 ^d^
PP3	460 ± 83 ^de^	91 ± 6 ^d^	55 ± 1 ^d^	144 ± 21 ^c^
PP4	1257 ± 38 ^c^	75 ± 5 ^d^	52 ± 1 ^d^	856 ± 25 ^d^
Wheat Mix	300 ± 11 ^ef^	453 ± 2 ^b^	91 ± 0 ^b^	251 ± 8 ^a^
VWG	211 ± 23 ^f^	450 ± 2 ^b^	91 ± 1 ^d^	156 ± 14 ^cd^
Soy Mix	1626 ± 94 ^b^	56 ± 31 ^d^	51 ± 1 ^d^	403 ± 10 ^d^
SPC	320 ± 47 ^e^	552 ± 0 ^a^	70 ± 0 ^a^	580 ± 47 ^d^

## Data Availability

Data are contained within the article.

## References

[1] Estell M., Hughes J., Grafenauer S. (2021). Plant Protein and Plant-Based Meat Alternatives: Consumer and Nutrition Professional Attitudes and Perceptions. Sustainability.

[2] 2019 State of the Industry Report: Plant-Based Meat, Eggs, and Dairy. https://gfi.org/resource/plant-based-meat-eggs-and-dairy-state-of-the-industry-report/.

[3] Plattner B.J. (2022). Impact of Plant Protein Functionality and Extrusion Conditions on Texture of High Moisture Meat Analogs (HMMAs). Master’s Thesis.

[4] Webb D., Dogan H., Li Y., Alavi S. (2023). Use of Legume Flours and Fiber for Tailoring Structure and Texture of Pea Protein-Based Extruded Meat Alternatives. J. Food Sci..

[5] Kazir M., Livney Y.D. (2021). Plant-Based Seafood Analogs. Molecules.

[6] Plant-Based Meat Market Size, Share & Trends Analysis Report by Source (Soy, Pea), by Product (Burgers, Sausages), by Type (Chicken, Fish), by End-User (Retail, HORECA), by Storage, by Region, and Segment Forecasts, 2020–2027. https://www.grandviewresearch.com/industry-analysis/plant-based-meat-market.

[7] Forecast Value of the Pea Protein Market Worldwide from 2017 to 2027. https://www.prnewswire.com/news-releases/pea-protein-market-size-is-expected-to-reach-2-9-billion-by-2027--exclusive-report-by-marketsandmarkets-301596804.html#:~:text=Pea%20Protein%20Market%20size%20is,Exclusive%20Report%20by%20MarketsandMarkets%E2%84%A2.

[8] Poore J., Nemecek T. (2018). Reducing Food’s Environmental Impacts through Producers and Consumers. Science.

[9] Alternative Proteins: The Race for Market Share Is On. https://www.mckinsey.com/industries/agriculture/our-insights/alternative-proteins-the-race-for-market-share-is-on#.

[10] Webb D., Li Y., Alavi S. (2023). Chemical and Physicochemical Features of Common Plant Proteins and their Extrudates for Use in Plant-Based Meat. Trends Food Sci. Technol..

[11] Boukid F., Rosell C.M., Castellari M. (2021). Pea Protein Ingredients: A Mainstream Ingredient to (Re)Formulate Innovative Foods and Beverages. Trends Food Sci. Technol..

[12] Kornet R., Veenemans J., Venema P., van der Goot A.J., Meinders M., Sagis L., van der Linden E. (2021). Less Is More: Limited Fractionation Yields Stronger Gels for Pea Proteins. Food Hydrocoll..

[13] Gao Z., Shen P., Lan Y., Cui L., Ohm J.-B., Chen B., Rao J. (2020). Effect of Alkaline Extraction PH on Structure Properties, Solubility, and Beany Flavor of Yellow Pea Protein Isolate. Food Res. Int..

[14] García Arteaga V., Kraus S., Schott M., Muranyi I., Schweiggert-Weisz U., Eisner P. (2021). Screening of Twelve Pea (*Pisum Sativum* L.) Cultivars and Their Isolates Focusing on the Protein Characterization, Functionality, and Sensory Profiles. Foods.

[15] Sirtori E., Isak I., Resta D., Boschin G., Arnoldi A. (2012). Mechanical and Thermal Processing Effects on Protein Integrity and Peptide Fingerprint of Pea Protein Isolate. Food Chem..

[16] Lam A.C.Y., Can Karaca A., Tyler R.T., Nickerson M.T. (2018). Pea Protein Isolates: Structure, Extraction, and Functionality. Food Rev. Int..

[17] Day L. (2013). Proteins from Land Plants—Potential Resources for Human Nutrition and Food Security. Trends Food Sci. Technol..

[18] Food Data Central Agricultural Research Service. United State Department of Agriculture. https://fdc.nal.usda.gov/index.html.

[19] Anderson R.A., Conway H.F., Peplinski A.J. (1970). Gelatinization of Corn Grits by Roll Cooking, Extrusion Cooking and Steaming. Starch—Stärke.

[20] Brishti F., Zarei M., Muhammad K., Ismail-Fitry M., Shukri R., Saari N. (2017). Evaluation of the Functional Properties of Mung Bean Protein Isolate for Development of Textured Vegetable Protein. Int. Food Res. J..

[21] Shahsavani Mojarrad L., Rafe A., Sadeghian A., Niazmand R. (2017). Effects of High Amylose Corn Starch and Microbial Transglutaminase on the Textural and Microstructural Properties of Wheat Flour Composite Gels at High Temperatures. J. Texture Stud..

[22] Webb D., Plattner B.J., Donald E., Funk D., Plattner B.S., Alavi S. (2020). Role of Chickpea Flour in Texturization of Extruded Pea Protein. J. Food Sci..

[23] Kearns J.P., Rokey G.J., Huber G.R., Applewhite T.H. (1989). Extrusion of Texturized Proteins. Proceedings of the World Congress on Vegetable Protein Utilization in Human.

[24] AMSA (2015). Research Guidelines for Cookery, Sensory Evaluation, and Instrumental Tenderness Measurements of Meat.

[25] Osen R., Toelstede S., Wild F., Eisner P., Schweiggert-Weisz U. (2014). High Moisture Extrusion Cooking of Pea Protein Isolates: Raw Material Characteristics, Extruder Responses, and Texture Properties. J. Food Eng..

[26] Urade R., Sato N., Sugiyama M. (2017). Gliadins from Wheat Grain: An Overview, from Primary Structure to Nanostructures of Aggregates. Biophys. Rev..

[27] Jones O.G. (2016). Recent Advances in the Functionality of Non-Animal-Sourced Proteins Contributing to Their Use in Meat Analogs. Curr. Opin. Food Sci..

[28] Florence O. (2012). Uruakpa Gelling Behavior of Plant Proteins and Polysaccharides in Food Systems. JFSE.

[29] Mession J.-L., Chihi M.L., Sok N., Saurel R. (2015). Effect of Globular Pea Proteins Fractionation on Their Heat-Induced Aggregation and Acid Cold-Set Gelation. Food Hydrocoll..

[30] McClements D.J., Grossmann L. (2021). The Science of Plant-Based Foods: Constructing next-Generation Meat, Fish, Milk, and Egg Analogs. Compr. Rev. Food Sci. Food Saf..

[31] Aydemir L.Y., Yemenicioğlu A. (2013). Potential of Turkish Kabuli Type Chickpea and Green and Red Lentil Cultivars as Source of Soy and Animal Origin Functional Protein Alternatives. LWT—Food Sci. Technol..

[32] Renard D., Lefebvre J. (1992). Gelation of Globular Proteins: Effect of PH and Ionic Strength on the Critical Concentration for Gel Formation. A Simple Model and Its Application to Beta-Lactoglobulin Heat-Induced Gelation. Int. J. Biol. Macromol..

[33] Nicolai T., Chassenieux C. (2019). Heat-Induced Gelation of Plant Globulins. Curr. Opin. Food Sci..

[34] Tanger C., Engel J., Kulozik U. (2020). Influence of Extraction Conditions on the Conformational Alteration of Pea Protein Extracted from Pea Flour. Food Hydrocoll..

[35] O’Kane F.E. (2004). Molecular Characterisation and Heat-Induced Gelation of Pea Vicilin and Legumin. Ph.D. Thesis.

[36] Beck S.M., Knoerzer K., Arcot J. (2017). Effect of Low Moisture Extrusion on a Pea Protein Isolate’s Expansion, Solubility, Molecular Weight Distribution and Secondary Structure as Determined by Fourier Transform Infrared Spectroscopy (FTIR). J. Food Eng..

[37] Karkle E.L., Alavi S., Dogan H. (2012). Cellular Architecture and Its Relationship with Mechanical Properties in Expanded Extrudates Containing Apple Pomace. Food Res. Int..

[38] Carvalho C.W.P., Takeiti C.Y., Onwulata C.I., Pordesimo L.O. (2010). Relative Effect of Particle Size on the Physical Properties of Corn Meal Extrudates: Effect of Particle Size on the Extrusion of Corn Meal. J. Food Eng..

[39] Webb D.M. (2021). Physicochemical Properties of Pea Proteins, Texturization Using Extrusion, and Application in Plant-Based Meats. Master’s Thesis.

[40] de Mesa N.J.E., Alavi S., Singh N., Shi Y.-C., Dogan H., Sang Y. (2009). Soy Protein-Fortified Expanded Extrudates: Baseline Study Using Normal Corn Starch. J. Food Eng..

[41] Kim T. (2018). Texturization of Pulse Proteins: Peas, Lentils, and Faba Beans. Ph.D. Thesis.

